# Involvement of the Intrinsic/Default System in Movement-Related Self Recognition

**DOI:** 10.1371/journal.pone.0007527

**Published:** 2009-10-21

**Authors:** Roy Salomon, Rafael Malach, Dominique Lamy

**Affiliations:** 1 Department of Psychology, Tel Aviv University, Tel-Aviv, Israel; 2 Department of Neurobiology, Weizmann Institute of Science, Rehovot, Israel; Cuban Neuroscience Center, Cuba

## Abstract

The question of how people recognize themselves and separate themselves from the environment and others has long intrigued philosophers and scientists. Recent findings have linked regions of the ‘default brain’ or ‘intrinsic system’ to self-related processing. We used a paradigm in which subjects had to rely on subtle sensory-motor synchronization differences to determine whether a viewed movement belonged to them or to another person, while stimuli and task demands associated with the “responded self” and “responded other” conditions were precisely matched. Self recognition was associated with enhanced brain activity in several ROIs of the intrinsic system, whereas no differences emerged within the extrinsic system. This self-related effect was found even in cases where the sensory-motor aspects were precisely matched. Control conditions ruled out task difficulty as the source of the differential self-related effects. The findings shed light on the neural systems underlying bodily self recognition.

## Introduction

The division between the self and the external environment is a fundamental aspect of our psychological life [1], [2]. The question of the nature and status of self-related processing has been the focus of heated debate in the history of philosophy and psychology [1], [3]–[5] and more recently in brain research [6]–[10]. The multi-faceted nature of the concept of self poses a major challenge in the search for the neural correlates of self-related processing. Basic theoretical distinctions have been developed by philosophers and further refined by recent research. The physical self (James,1890) or ‘proto-self’ [11], [12] refers to a preconscious representation of the self in the sensory and motor domains. The mental self [1] or ‘minimal self’ [13], [14] refers to “a consciousness of oneself as an immediate subject of experience, unextended in time” [15]. The “spiritual” [1], “autobiographical” [11] or “narrative” [15] self extends the representation of the self in time. Further functionally based distinctions have burgeoned in the recent literature and include the facial self [16]–[19], emotional self [20], [21], verbal self [22], spatial self [23], [24] and social self [25], [26].

Processing of the diverse aspects of the self draws on information derived from various sensory and cognitive processes associated with separate brain networks. Yet, phenomenologically, the sense of self is unified [15] and the brain integrates the different aspects of the self into a single cohesive concept. A fundamental question is whether the sense of self is represented in cortical regions that integrate and read-out “lower level” sensory-motor information, or if it is represented in a specialized, unitary system. The current literature points to a convergence of self- related activity in the cortical midline regions, specifically the prefrontal dorsal and ventral medial cortex as well as the posterior medial and precuneus regions [10 for a review], which was observed using different operationalizations of the self [6], [9], [27], [28].

Recent fMRI research has revealed a new fundamental cortical subdivision into two global systems. One system, which we have called the “extrinsic system”, [29] encompasses all the sensory-motor areas engaged with processing and acting on information derived from the outside environment. This system shows high levels of inter- and intra-subject correlation in response to natural stimuli.[29], [30]. It includes the occipital, parietal and temporal primary and secondary sensory regions, as well as the frontal motor and premotor regions. The second system, which we have called the “intrinsic system” [29], [31], shows task-related deactivations, that is, activity reduction during tasks involving processing of external stimuli [32], [33], such that its activity is highly anticorrelated with that of the extrinsic system. The intrinsic system substantially overlaps the system described by Raichle and colleagues as the default mode network [6], [34], [35]. It includes the prefrontal medial and superior frontal cortex, the posterior medial part of the cingulate gyrus and precuneus, and the bilateral inferior parietal cortex. Whereas the extrinsic system has been extensively investigated and the stimulus types processed by its different regions are fairly well characterized, the functional organization of the intrinsic system is much less understood. To date, it has been only broadly associated with processing of information derived from the organism itself [6], [33]. Accordingly, a growing body of data has implicated this system in various “inward”-oriented tasks, such as mental-state attribution [36], perspective taking [37], daydreaming [38], emotional processing [39] and theory of mind [14].

It is noteworthy that selective activation of regions of the intrinsic system has been reported in recent research specifically aimed at describing the neural correlates of self-related processing ( [9] for a review). In these studies, the “self” condition was operationalized using diverse self-referential activities such as reflection on personality traits and physical appearance of self vs. other [27], retrieval of personality trait adjectives [40], evaluation of personality traits as self descriptive [41], first-person perspective taking [24], introspection [8], voluntary decision [42], and facial self recognition [19], [36].

However, these studies typically included differences in stimulus or task conditions that were confounded with the self vs. other manipulation. Some [6], [8] contrasted an emotional valence task (self condition) with a perceptual decision task (non-self condition). Other studies required subjects to imagine different mental perspectives [43], [44], or to make judgements about character traits of self vs. other people [27]; [45]. Others employed the same task in the “self” and “other” conditions but used different stimuli in the two conditions. For instance, in self-face recognition tasks, pictures of self were contrasted with pictures of either unfamiliar [19] or famous [36] people. Similar limitations apply to studies using point-of-view tasks [24], [37].

In the current study we explored judgments on a fundamental and concrete level of self processing often termed the ‘minimal self’ [13]. The minimal self includes embodiment or body-ownership (the sense of our consciousness residing in a body) and agency (the sensation of willed control over the movements of that body), which under normal conditions are in complete accord, such that “my body does what I will it to do”. An important advantage of this operationalization of the self is that it refers to a primary level of self schema that is less likely to be confounded with individual or cultural differences [46]. Accordingly, the formation of the sense of agency and embodiment precedes developmentally the more abstract levels of self [15], [47]. In the current experiment we manipulated the sense-of-embodiment aspect of the minimal self by requiring the subjects to attribute viewed hand movements to themselves or to another person and tested the extent to which agency affected performance on this discrimination.

**Figure 1:**
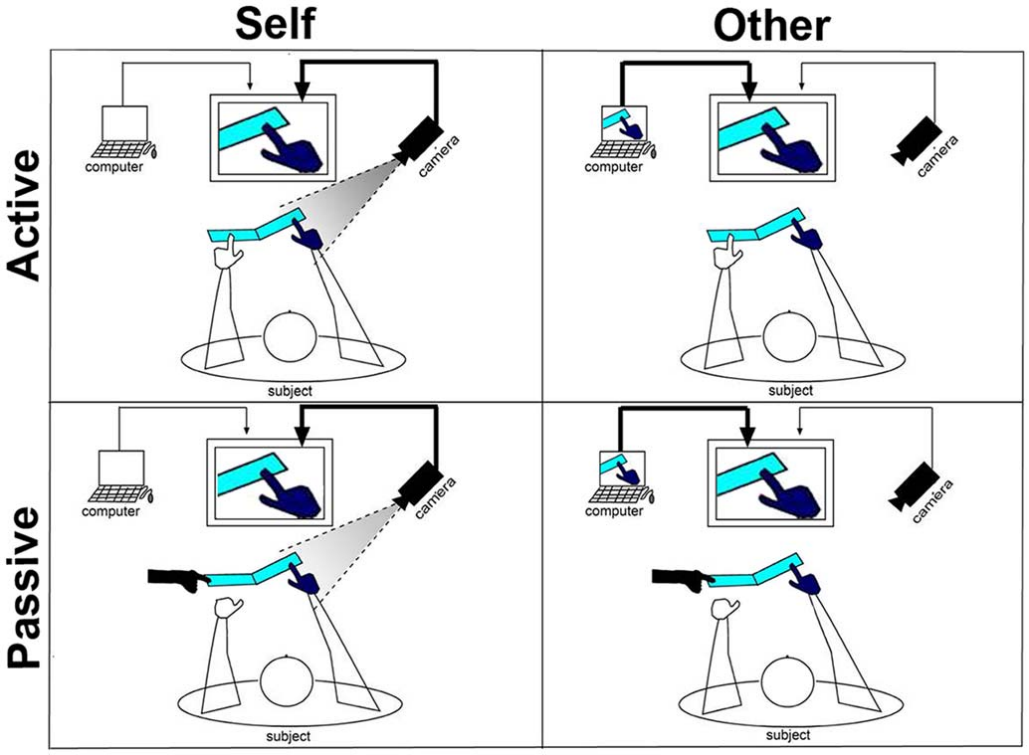
2x2 factorial design of the experiment. *Left panels*: “View Self” condition, in which the subjects saw their own right hands in a live movie, that is, a movement than was perfectly synchronized with their own. *Right panels*: “View Other” condition, in which the subjects saw the same hands but in a pre-recorded movie, such that there were small timing differences between the viewed and the executed movements. *Upper panels*: “Active” condition, in which the lever that lifted the subjects' right hands was pressed by the subjects' left hands. *Lower panels*: “Passive” condition, in which the lever was pressed by the experimenter. The subjects' right hands were gloved in order to prevent any attempt to distinguish between the view-self and view-other conditions based on morphological differences.

**Figure 2:**
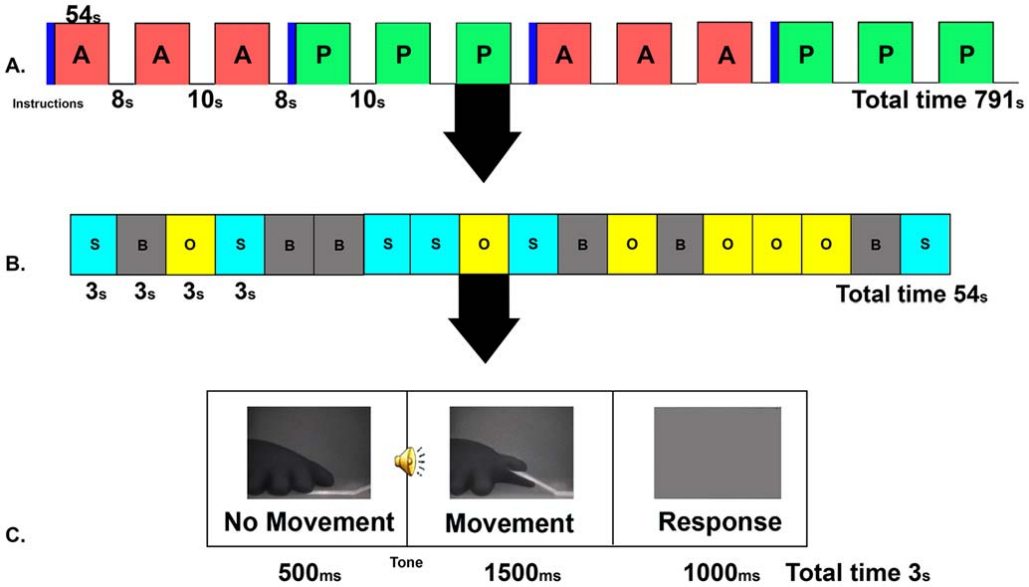
Combined blocked and event-related experimental paradigm. A. *Blocked design*. Red epochs represent the active blocks and green epochs represent the passive blocks. The blue bars represent the instructions given before each block triplet of the same type. B. *Event-related design within each block*. Light blue bars represent view-self events and yellow bars represent view-other events (motion trials). Gray bars represent fixation events. C. *Sequence of events making up motion trials*. Subjects viewed a hand at rest for 500 ms. Then, following an auditory cue, the lever pressing sequence started and the moving hand was viewed for 1,500 ms. A 1,000-ms interval followed during which the screen went blank and subjects responded whether the viewed hand had been their own or someone else's hand.

We employed an experimental paradigm adapted from [48] and depicted in Figures 1 & 2. This paradigm has the advantage that self-recognition can be manipulated while all external sensory-motor aspects are tightly matched. Subjects viewed the motion of a gloved hand on a screen, either their own hand in a live movie (“View Self”) or the same hand but in a pre-recorded movie (as subjects were led to believe that they would be viewing another person's hand and the effectiveness of this manipulation was validated in post experimental debriefing we have termed this as “View Other”). As the movement was highly constrained, the two conditions were precisely matched in terms of the sensory stimuli and motor responses, and differed only in subtle timing variations between the viewed and the executed movement. Based on such differences, subjects had to report whether the viewed hand was their own or someone else's. In addition, the hand's motion was induced either by the subject's other hand (“Active motion”) or by the experimenter (“Passive motion”) using a lever. Importantly, in the present design the stimuli and task demands associated with the “responded self” and “responded other” conditions were closely matched. We expected to observe higher levels of brain activity within the intrinsic system when subjects judged the viewed hand to be their own (“responded self” condition) as opposed to someone else's (“responded other” condition). Our results confirm this prediction. Control analyses ruled out the possibility that factors other than the self vs. other distinction, namely arousal, task difficulty or synchronicity between the viewed and executed movement, might account for the observed differences. The present data provide the first evidence of enhanced activation of the human intrinsic system in self vs. other conditions that entail identical stimuli and response parameters.

## Methods

### Participants

Eleven healthy subjects (ages 22–32, right-handed as measured by a laterality quotient >50 on the Edinburgh handedness inventory[49]) participated in the experiment. Subjects had normal or corrected to-normal vision. The Tel-Aviv Sourasky Medical Center approved the experiment and issued an according Helsinki agreement. All subjects gave written informed consent for both the experiment and the imaging procedure. Four additional subjects were removed from the analysis due to movement artifacts.

### Experimental Paradigm

The movement was a passive elevation of the subject's right index finger by a lever (angle 135°), which allowed separating the action from its somatic effect. The lever was pressed either by the subject's own left hand (‘Active’ condition), or by the experimenter (‘Passive’ condition). Pressing the lever was prompted by an auditory cue delivered via headphones. During the movement, subjects viewed video movies of their own right hand either live (“view self”, henceforth “VS” condition) or pre-recorded during the training session (“view other”, henceforth “VO” condition) (see Figure. 2). The movies were relayed by an LCD projector (Epson MP 7200) onto a tangent screen positioned in front of the subjects' foreheads and viewed through a tilted mirror placed above their heads. In all conditions only the right hand and right side of lever were visible on the screen. Video images were displayed by means of custom-built software.

Subjects lay prone in the fMRI scanner at Tel-Aviv Sourasky Medical Center, with a pillow placed on their chests and a wooden board over the abdomen area. The pillow served to occlude direct view of their hands. Importantly, the subjects wore dark woollen gloves so as to preclude the possibility that any physical cues might promote self-hand recognition. The fingers of their right hands were fixed to the table using pre-placed Velcro strips, except for the index finger which was fixed to one end of the lever. Their left hands were placed on the table with the left index finger placed on the other end of the lever.

Subjects were told that the experiment tested self-recognition ability based on motion. While the experimenter explained the task, the subjects viewed a sample video that depicted the hand of another subject. This procedure was aimed at strengthening the subjects' belief that the View Other condition indeed consisted of videos of other people's hands. Then the subjects completed a practice block (9 active trials and 9 passive trials), during which 16 video clips of each subject's movement were recorded. Of these, one representative active and one representative passive clips were selected by the experimenter for use in the experiment as the stimuli of the ‘view other’ condition.

The experiment followed the practice trials. After they completed the experiment the subjects were asked to fill out a personal questionnaire and the Edinburgh handedness inventory [49]. The subjects were interviewed in order to assess the perceived difficulty of the experiment in the two conditions, the effectiveness of the deception procedure designed to have them believe that their own pre-recorded hand belonged to someone else, and their confidence regarding their responses and to uncover any special strategy they might have used in selecting their responses. The subjects were then fully debriefed and thanked for their participation.

### Experimental Design

Conditions of movement authorship (Active/Passive) were run in sequences of three blocks per condition, with condition order counterbalanced between subjects. Conditions of identity (VS/VO) were pseudo-randomly mixed within blocks, together with a “fixation” condition, consisting of the presentation of a fixation sign in the center of an otherwise blank screen. The latter condition was added so as to allow event-related deconvolution.

There were 12 blocks altogether, 6 for the active condition and 6 for the passive condition. Each block contained 18 trials (6 per condition – VS/VO/fixation). Each trial lasted 3000 msec. In the VS and VO conditions, a still image of the hand appeared for 500 ms. Then a 100-ms audio cue was onset at the same time as a 1500-ms movie of the hand, during which subjects had to decide whether they were viewing their own hands or someone else's. On each trial, subjects responded by moving one of their feet, one foot if they judged that the hand they viewed was their own and the other foot if they judged that the hand was someone else's. Response-to-foot assignment was counterbalanced between subjects. A 1000-ms response interval followed during which the screen went blank. In the fixation condition, the fixation display was presented for 3000 ms.

There were 216 trials in total, such that the experimental blocks lasted 10 min 48 sec. There was a short break of either 8 or 10 sec after each block, with an additional 5-sec break every 3 blocks, during which the task was changed to a different condition of movement authorship. All trials were monitored online by an experimenter who inspected both the subject's movement and the movement shown on the screen to ensure that the timing of the subject's response and the viewed action were synchronized. Any trials judged by the experimenter to be deviant were excluded from the analysis (4% of all trials).

### Imaging Procedure

Subjects were scanned on a 3 Tesla Signa Horizon LX 8.25 GE scanner equipped with a standard birdcage head coil. Blood oxygen level dependent (BOLD) contrast was obtained with gradient echo echo-planar imaging (EPI) sequence (TR, 1000; TE, 30; flip angle, 90°; field of view, 24×24 cm2; matrix size, 80×80). The scanned volume included 16 nearly axial slices of 6-mm thickness and 1-mm gap, so as to cover the entire cortical surface of the brain. T1-weighted high-resolution (1.1×1.1 mm) anatomical images and a whole-brain spoiled gradient (SPGR) sequences were acquired for each subject to allow accurate cortical segmentation and reconstruction, and volume-based statistical analysis. The cortical surface was reconstructed from the three-dimensional SPGR scan and was then unfolded and flattened. The obtained activation maps were superimposed on the unfolded and inflated cortices.

### Data Analysis

fMRI data were analyzed with the “BrainVoyager” software package (Brain Innovation, Masstricht, Netherlands) and with complementary in-house software. The cortical surface in a Talairach coordinate system [50] was reconstructed from the 3D-spoiled gradient echo scan for each subject. The obtained activation maps were superimposed on the unfolded cortex. Preprocessing of functional scans included 3D motion correction and filtering out of low frequencies up to twelve cycles per experiment (slow drift). Statistical mapping was based on the General Linear Model [51]. Our analysis consisted of a multiple regression with a regressor for each condition in the experiment and assuming a hemodynamic lag of 5–6 s. Predictors were convolved with a standard two-gamma HRF waveform. The data was spatially smoothed with a Gaussian filter of full width of half maximum value of 4 mm.

The analysis was performed independently for the time course of each individual voxel.

The multi-subject maps were obtained using the screening for partial conjunction analysis method suggested in Heller [52] to create informative group maps. In order to obtain activation maps showing the voxels activated by at least u subjects while controlling for the FDR, the following analysis was done. First, we combined the p-values per voxel using equation (6)
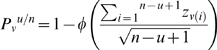
and (7) [52]. 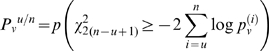



Second, we applied the BH procedure on the resulting combined p-value map. This analysis was repeated for all values of u = 1,…,n, and the n activation maps were superimposed on the same display. The multi-subject functional maps were projected on an inflated or unfolded Talairach normalized brain.

### Definitions of ROIs

ROIs were defined both functionally and anatomically based on the regions showing negative activity on the all tasks-versus-fixation contrast. This was done on a subject-by-subject basis with a minimum p value of 0.01, corrected. Each ROI was defined with the constraint that it contained at least 200 contiguous voxels and overlapped the regions of the intrinsic system as discussed in [29]. Six ROIs were selected for analysis: medial prefrontal cortex (MPFC), right and left superior and prefrontal cortex (RSFC & LSFC), precuneus (PCUN) and right and left posterior parietal cortex (RIPC & LIPC). Talairach coordinates were determined for the center of each ROI. Time courses for each of the ROIs were extracted for each subject and then averaged. Peak activations were averaged for each condition and compared using Student's t tests.

Extrinsic ROIs were selected in a similar manner using the regions showing strongest positive activations in the all task vs. baseline condition. These included bilateral lateral occipital cortex, bilateral inferior occipital cortex, dorsal medial prefrontal cortex, the bilateral post central sulcus, bilateral superior intraparietal sulcus, left post central gyrus and the superior part of the right lateral sulcus.

## Results

### Behavioral results

**Figure 3:**
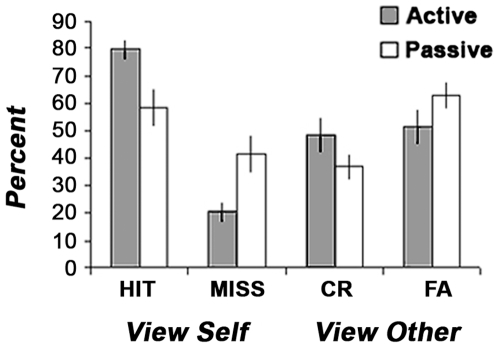
Signal Detection analysis of subjects' self recognition performance. Proportion of view-self trials in which subjects correctly identified their hands (Hits) vs. incorrectly judged them to belong to someone else (Misses) and proportion of the view-other trials in which the subjects correctly judged the viewed hands to be someone else's (CR - Correct Rejections) vs. incorrectly judged them to be their own (FA - False Alarms). Note that there were more errors in the passive relative to the active condition, indicating that the task was more difficult when subjects were not the authors of the viewed movement.

**Figure 4:**
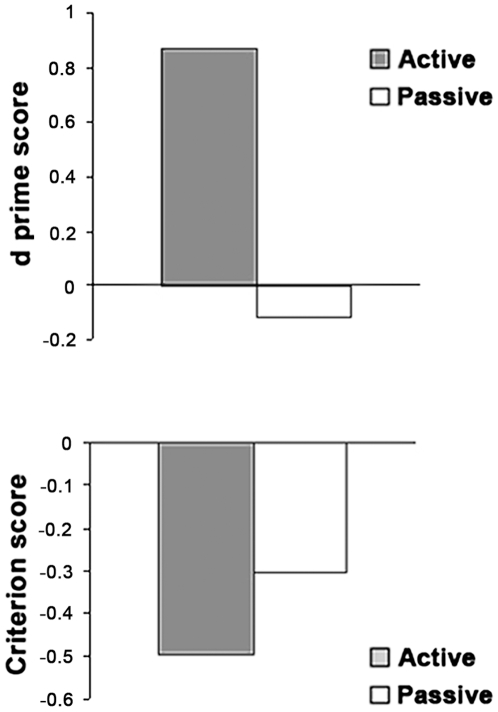
Signal Detection analysis of behavioural responses by conditions of movement ownership. *Upper panel*: Sensitivity (d') in the active vs. passive condition. *Lower panel*: Criterion or response bias in the active vs. passive condition. Subjects were better at making the self vs. other discrimination (p<.05) and tended to judge the viewed hand to be their own in the active relative to the passive condition (p = .06).

Behavioral self-recognition performance was assessed based on Signal Detection Theory [53]. Mean proportions of Hits (self hand correctly attributed to self), Misses (self hand incorrectly attributed to other), Correct Rejections (other's hand correctly attributed to other) and False alarms (other's hand incorrectly attributed to self) are presented separately for the “Active” and “Passive” conditions in Figure 3. Our findings are consistent with the results reported in the original experiment by [48] although overall accuracy was somewhat lower, probably due to increased difficulty in performing the task in the fMRI scanner. The relatively high proportion of false alarms attests of the difficulty in discriminating between self and other motion in the absence of physical stimulus differences and based on timing differences alone. Sensitivity measured as d' was higher in the active than in the passive condition (Figure 4, upper panel), Wilcoxon t(10) = 2.48, p = 0.01. While it was different from 0 in the active condition, indicating that subjects were able to discriminate between self and other's motion (d' = 0.87, Wilcoxon t(10) p<0.01) it was null in the passive condition, (d' = −0.12, Wilcoxon t(10), p = 0.92). In addition, subjects showed a self-attribution bias, that is, more readiness to respond “self” than to respond “other”, a bias that tended to be higher in the active than in the passive condition (c = −0.50. vs. c = −0.30, Wilcoxon t(10)  = 1.7, p = 0.06, see Figure 4, lower panel).

### fMRI data – Movement attribution to self vs. other

**Figure 5:**
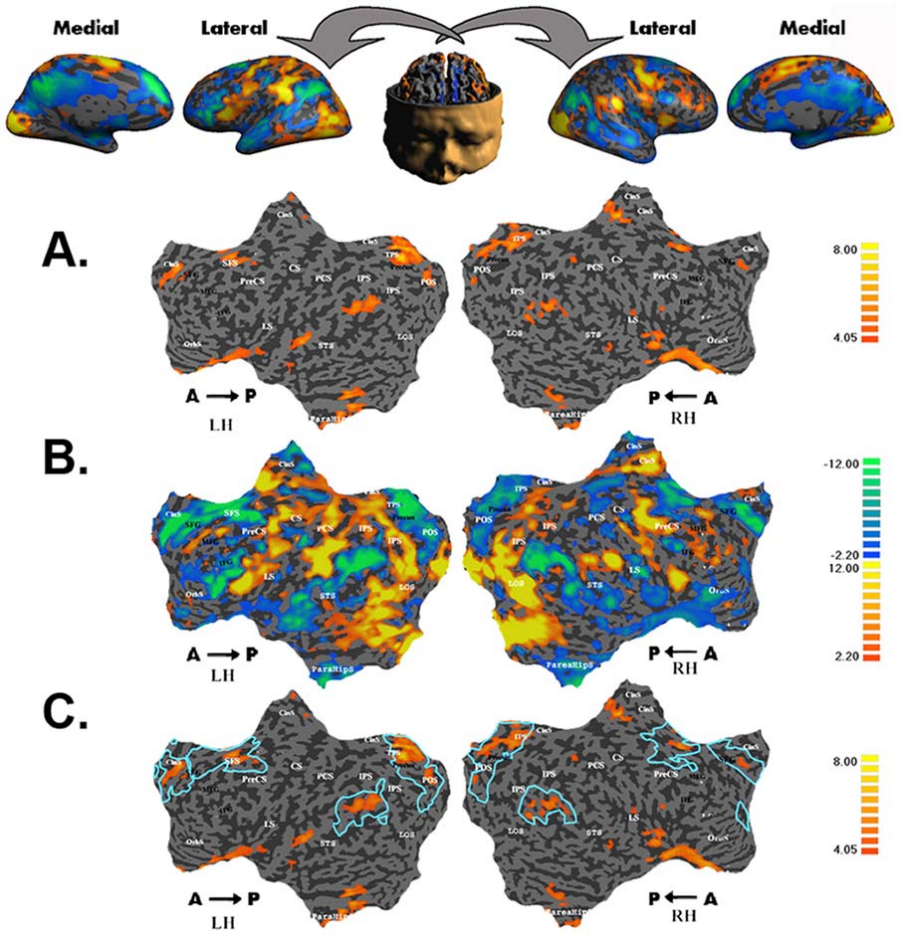
Overlap between deactivated regions of the intrinsic system and regions activated by self recognition. Single-subject (MZ) maps. A. BOLD activations for responded self vs. responded other conditions (self > other). B. BOLD activations for all tasks vs. fixation events (all tasks > fixation). Regions of the extrinsic system are shown in red-orange, and regions of the intrinsic system in blue-green. C. Overlap between the regions activated by self recognition (responded self vs. responded other conditions, self > other) and the regions deactivated by the all task vs. fixation comparison (intrinsic system). CS, central sulcus; SFG, superior frontal gyrus; SFS, superior frontal sulcus; ACC, anterior cingulate cortex; pCgS, paracingulate sulcus; STS, superior temporal sulcus; IFS, inferior frontal sulcus; IFG, inferior frontal gyrus; IPS, intraparietal sulcus; IPC, inferior parietal cortex; LS, lateral sulcus; PCUN, precuneus; LOC, lateral occipital complex; LH, left hemisphere; RH, right hemisphere.

We contrasted trials in which participants subjectively experienced ownership of the viewed hand (“responded self” condition) with trials in which they judged the hand to belong to someone else (“responded other” condition), regardless of which physical stimulus (self or other) was actually presented. The “responded self” and “responded other” conditions were identical in terms of stimuli, motor output and cognitive task, and thus differed only in the subjective attribution of ownership of the viewed hand. The results from subject MZ are presented in Figure 5A. The regions that were significantly more activated in the “responded self” than in the “responded other” condition were the medial prefrontal cortex, superior frontal cortex bilaterally, parahippocampal gyrus, ventral region of the lateral sulcus, inferior parietal cortex and precuneus, bilaterally.

In order to map the extrinsic and intrinsic systems in the current study, we contrasted conditions that differed markedly in the amount of external demands they involved, namely, all tasks (active + passive) vs. fixation. The map for subject MZ is shown in Figure 5B. Regions that were more activated during task blocks than during fixation blocks include the known regions of the extrinsic system (low- and high-level visual areas, sensory-motor regions and premotor regions). Regions that were more deactivated during task blocks than during fixation blocks include the medial prefrontal and precuneus regions, bilateral superior frontal cortex, inferior parietal regions and temporal regions.

These maps were used on a subject-by-subject basis to map the ROIs of the intrinsic system as described in the methods. The ROIs closely corresponded to the regions identified as belonging to the intrinsic system in previous studies [29]. Most importantly and as predicted, there was considerable overlap between the regions of responded- self vs. responded-other activations and the selected ROIs of the intrinsic system as shown in Figure 5C. Note that the other foci of activity, namely, in the parahippocampal gyrus and medial aspect of the lateral sulcus, also overlapped with regions found to activate the intrinsic system in the present study. However, because these regions have not been consistently associated with the intrinsic system in previous studies, they were not considered as ROIs here.

**Figure 6:**
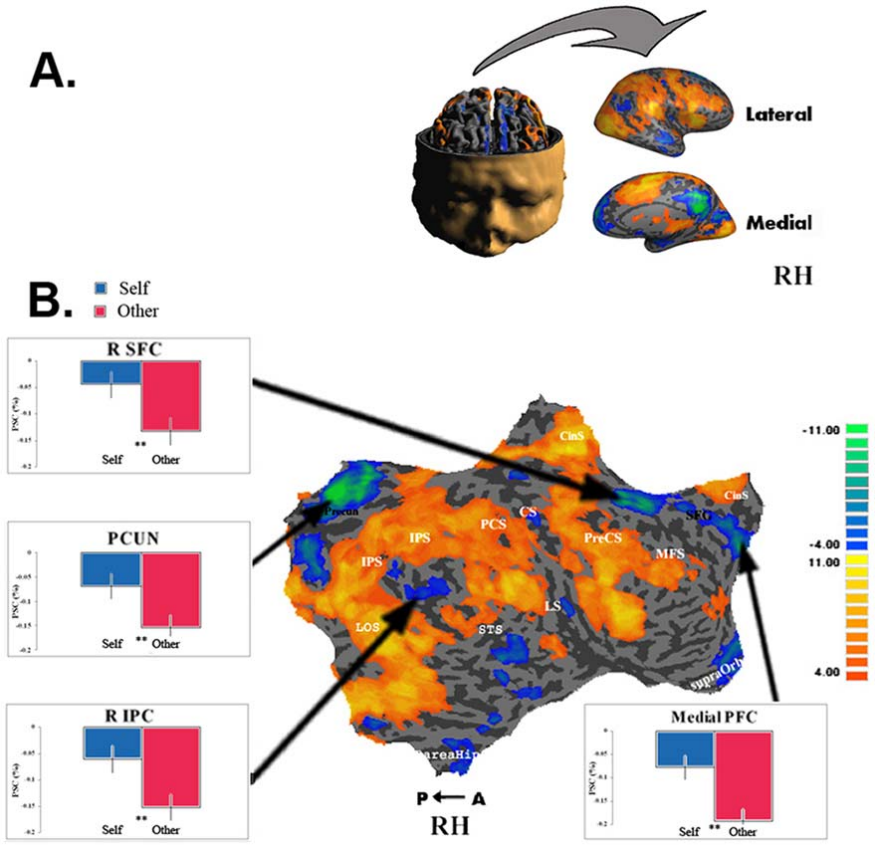
Intrinsic ROIs and Self Recognition – Right Hemisphere. A. Folded and inflated views of RH. B. Conjunction map of all tasks vs. fixation contrast. Multi-subject analysis (N = 11) testing whether at least one contrast activated the region in at least u subjects with FDR <0.05. The intensity represents the minimum number of subjects for whom the region was activated, ranging from at least 4 subjects (orange) to 11 subjects (yellow). Graphs show multi-subject average BOLD activations of ROIs for responded self and responded other conditions. Intrinsic ROIs showed higher levels of activation in the responded self than in the responded other condition. Error bars represent SEM. ** p<0.01.

Multi-subject data (N = 11) for the mapping of the extrinsic and intrinsic systems (all tasks vs. fixation) are shown in Figure 6. The multi-subject maps were obtained using the partial conjunction analysis method suggested by Heller [52] and described in the experimental procedures. Colors represent the numbers of subjects who showed significant activity in a given region with FDR level of p<0.05. For each ROI of the intrinsic system we averaged all subjects' BOLD signals for this region separately for the “responded self” and “responded other” conditions.

We then used one-tailed paired student t tests to compare the two conditions. In line with our predictions, activations in the ROIs of the intrinsic system showed consistent differences between the “responded self” and “responded other” conditions. While these differences were found in both hemispheres, the right hemisphere showed overall stronger effects. The data for the right hemisphere is presented here and the left hemisphere data can be viewed in Supplementary Figure S1.

In order to determine whether movement attribution and type of motion interacted with each other, we also conducted a 2X2 ANOVA (Self/Other X Active/Passive) for all intrinsic ROIs. The ANOVA showed no significant interactions between the two variables in any of the ROIs (all p>0.1). Furthermore the Active/Passive variable was not significant in any of the intrinsic ROIs (all p>0.1).

A similar 2X2 ANOVA (Self/Other X Active/Passive) was run to assess the effects of the experimental conditions in the extrinsic control regions. The results revealed that none of these regions showed significant differences between the “responded self”- and “responded other”-response conditions (all p>0.14). The comparison between active and passive motion conditions yielded significant differences in three regions: Activations were larger in the active relative to the passive condition in the dorsal medial prefrontal cortex (F 1,10 = 13.323, p = 0.0045), the right superior intraparietal sulcus (F 1,10 = 6.687, p = 0.0271) and the left superior intraparietal sulcus (F 1,10 = 6.393, p = 0.03). No significant interactions between self- vs. other-response and active vs passive motion were found (all p>0.05).

### Potential confounds

#### General arousal

**Figure 7:**
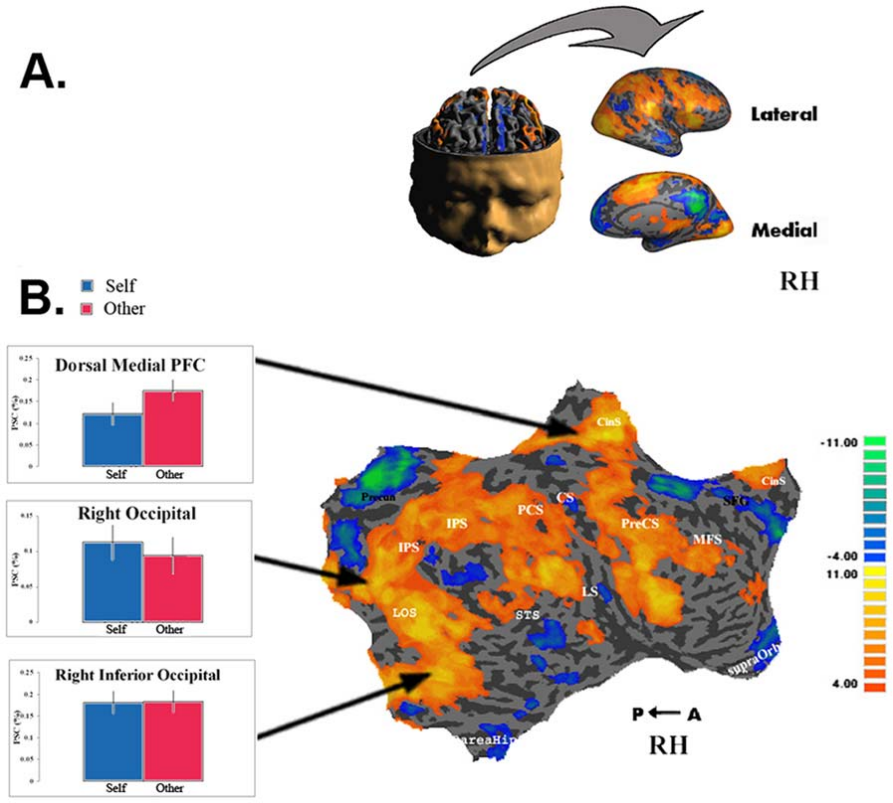
Extrinsic control regions and Self Recognition – Right Hemisphere. A. Folded and inflated views of RH. B. Conjunction map of all tasks vs. fixation contrast. Multi-subject analysis (N = 11) testing whether at least one contrast activated the region in at least u subjects with FDR <0.05. The intensity represents the minimum number of subjects for whom the region was activated, ranging from at least 4 subjects (orange) to 11 subjects (yellow). Graphs show multi-subject average BOLD activations of ROIs for responded self and for responded other conditions. Extrinsic control regions showed no difference in levels of activation between the responded self and responded other conditions. Error bars represent SEM.

To ensure that the higher levels of activity observed in the responded-self condition were specific to the intrinsic system, we sampled eleven control regions from the extrinsic system corresponding to the regions of highest activation in the all-task contrast and representing both sensory and motor systems. These included bilateral occipital cortex, bilateral inferior occipital cortex, dorsal medial prefrontal cortex, bilateral post central sulcus, bilateral superior intraparietal sulcus, left post central gyrus and the superior part of the right lateral sulcus. There was no differential activity between the “responded-self” and “responded-other” conditions in these regions of the right (Figure 7) and left (in Supplementary Figure S2) hemispheres (all p>0.1).

#### Task difficulty

Previous research has shown that the intrinsic system is more deactivated and the extrinsic system more activated in difficult relative to easy externally-oriented tasks [8], [54]. In our task, one might consider the “responded self” condition to have been easier than the “responded other” condition, as subjects showed a significant bias towards responding “self”. Thus, the smaller deactivation of the intrinsic system observed in the “self” relative to the “other” conditions might be due to the fact that the former condition was easier than the latter rather than differences related to self vs. other processing. However, several factors render this possibility improbable.

In previous work linking task difficulty and modulation of brain activity in the intrinsic system, the difficulty manipulations pertained to perceptual processing demands such as stimulus discrimination or stimulus presentation rate (e.g., [54], [55] rather than to response selection demands. Hence, the more pronounced deactivation of the intrinsic system was typically accompanied by higher activation of the extrinsic system, which reflects resource-demanding processing of external events [34]. In our task, only response selection requirements differed, as reflected by the response bias, whereas the perceptual discrimination required by the task was identical in the “responded self” and “responded other” conditions. In line with this argument, the activation pattern of the extrinsic system was no more pronounced in the responded other than in the responded self conditions.

**Figure 8:**
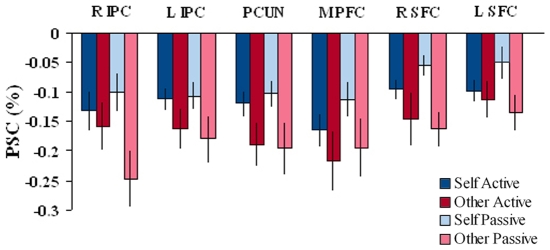
BOLD activations in the intrinsic ROIs by response type and movement authorship. Average BOLD activations in the ROIs for the four cells resulting from crossing the respond self vs. respond other conditions and the active vs. passive conditions. Note the similarity of responded self vs. responded other differences in the active and passive conditions. Error bars =  SEM.

Direct comparison of the passive and active conditions further argues against a role for task demands differences in driving the self vs. other effect. These conditions showed marked differences in terms of task difficulty, as indicated by the higher performance accuracy in the active relative to the passive condition (64.10% vs. 47.64%), and by the lower sensitivity (d' = 0.87 vs. d' = −0.12), respectively (see Figures 3 and 4). Yet, we observed no differential activations between the active and passive motion conditions in the intrinsic system, namely, in the IPC, SFC, Precuneus and Medial PFC (see Supplementary Figure S3). Furthermore the ROIs of the intrinsic system showed similar patterns of activation in the responded self vs. responded other conditions in both the active and passive conditions (see Figure 8).

#### Synchronicity

The only information on which subjects could rely to discriminate between self and other motion were slight violations of the synchronicity between actual and viewed movement. In fact, our procedure ensured that the final data set contained only trials in which these violations were so small that the experimenter who monitored the differences between the actual and viewed movement failed to notice them. Thus, the self vs. other manipulation was confounded with subtle differences in synchronicity.

**Figure 9:**
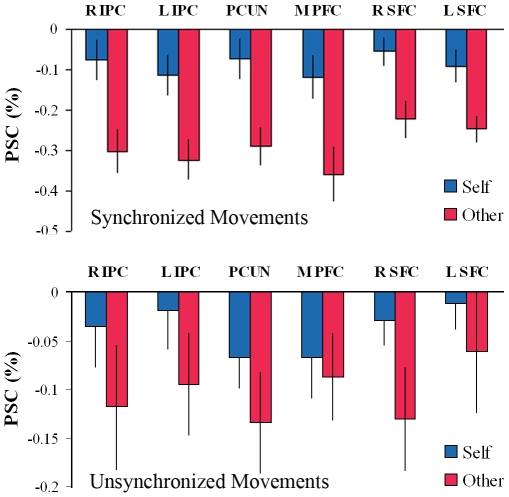
BOLD activations in the intrinsic ROIs by stimulus type. BOLD activations in intrinsic ROIs during passive blocks by response type, separately for synchronized movements -view-self condition (upper panel) and for unsynchronized movements – view other condition (lower panel). The subjective attribution of the viewed movement to self was associated with higher levels of BOLD activation in the intrinsic ROIs even though all external parameters were identical, and regardless of movement synchrony. Error bars =  SEM.

Note however that the critical comparison in the present study concerned brain activity associated with the subjective perception of the subjects (“responded self” vs. “responded other”) rather than with the objective stimulus that they viewed (“view self” vs. “view other”) and that was the variable confounded with synchronicity. The subjective perception corresponded to the objective stimulus only when the subjects responded correctly (i.e. when the seen and performed movements were synchronized and the subjects responded “self”, and when the movements were not synchronized and the subjects responded “other”), but the two could be distinguished by taking into account trials in which the subjects responses were incorrect. Accordingly, in order to address the possibility that synchronicity rather than perception of self vs. other accounted for our results, we compared activity in the intrinsic ROIs in the “responded-self” and “responded-other” conditions when the condition of synchronicity was the same in the two conditions. That is, we performed separate comparisons for trials in which the movements were synchronized (the subjects saw their hands online and either attributed the movement rightly to themselves or falsely to someone else) and for trials in which the movements were not synchronized (the subjects' saw a pre-recorded movement and either attributed it rightly to someone else or falsely to themselves). This comparison could be performed only for the passive condition because there were too few “miss” trials in the active condition to allow meaningful analysis. Our initial findings we replicated: the intrinsic ROIs showed higher activity in the “responded self” condition than in the “responded other” condition both for the view-self condition and for the view-other condition (see Figure 9). As the behavioral data suggest that recognition was at chance in the passive condition, these results show that the differential activation we observed in the ROIs of the intrinsic system are associated with the subjective attribution of movement to self since in this analysis all external factors were precisely matched in terms of movement authorship and synchronicity.

Furthermore, previous studies in which synchronicity alone was manipulated, with no embedded self- vs. other distinction, showed a pattern of brain activations very different from the one observed here. For instance, in a study by Leube and colleagues [56], subjects were aware of viewing their own movements throughout the experiment and determined whether or not a temporal delay was introduced in the visual feedback of these movements. Activations were found in the right posterior temporal lobe, which was not differentially activated by the “responded-self” vs. “responded-other” comparison in the present study and is also not traditionally associated with the intrinsic system.

### fMRI data - Agency

Our behavioral results showed a significant effect of agency on body ownership decisions, with better accuracy in the active trials. In an attempt to explore the neural correlates of this effect, we contrasted active blocks (in which the subjects induced their right-hand movements with their left hands) with passive blocks (in which the experimenter pressed the lever moving the subject's right hand finger). The results are shown in Supplementary Table S1.

The contrast yielded consistent effects in primary and secondary sensorimotor regions of the right hemisphere (pre and post central sulcus regions, p<0.003 uncorrected at the voxel level) and no significant effect in any of the ROIs of the intrinsic system. No significant interactions were found between response (self vs. other) and movement authorship (active vs. passive). Graphs of ROI activity in the active and passive conditions are presented in Supplementary Figure S3. The regions of significant activation in all conditions are presented in Supplementary Table S1.

## Discussion

### Self-related effects in the intrinsic system

Our findings show that regions of the intrinsic system were more active during attribution of a viewed movement to self than to another person (see Figure 6) even when the stimuli and task requirements pertaining to the “responded-self” and “responded-other” conditions were precisely identical (Figure 9). It is noteworthy that regardless of the condition (“responded-self” or “responded-other”), the signal measured in the intrinsic system was consistently below the baseline fixation level in the present study. Given our hypothesis that self-related processing should be counted among the various functional specializations of the intrinsic system, it may appear surprising that self attribution did not produce above baseline activity, but only a smaller level of deactivation relative to the other attribution. One possible explanation is that in addition to the self/other attribution, the present task involved processing video images, executing motor tasks and providing motor responses, all highly demanding extrinsic-related tasks that are expected to produce marked deactivation of the intrinsic system. Indeed, deactivation relative to “rest” baseline during processing and acting on information derived from the outside environment has been the most consistent functional “signature” of the intrinsic system, and has originally led to its definition as a “default” network (but see [57]). Given the sluggish nature of the fMRI signal, it is difficult to disentangle the negative-going extrinsic activation from the positive going self-related activation, such that the signal observed in the present study may be interpreted as being the sum of these opposite effects. The critical point is that the extrinsic aspects of the task were kept essentially identical while attribution of the viewed hand varied between “responded-self” and “responded-other” and was thus uniquely associated with the differential deactivations of the intrinsic system.

### Intrinsic Regions of Interest

#### Medial Prefrontal regions

Regions that comprise the midline intrinsic regions, namely, the medial ventral and dorsal prefrontal cortex and the posterior medial region including the posterior cingulate and precuneus regions, have been previously linked to self-related processing [37], [58]; [6], [9], [10]. Most of these studies used tasks involving emotional, memory or verbal domains rather than the motor domain [10], and therefore related predominantly to higher and more conceptual levels of self processing. In the present study, we investigated the minimal-self level of representation. The finding that the same regions are activated by a task from the sensorimotor domain provides strong converging evidence of task-independent self-related processing in these regions.

#### Dorsal Prefrontal regions

Findings from the recent literature suggest that dorsal prefrontal regions may be part of the fronto-parietal network associated with internal decisions and with monitoring for discrepancies between one's actions and the sensory outcomes of these actions. This network constitutes a fundamental aspect of the ‘minimal self' allowing discrimination of self from other. Accordingly, the left SFC was found to be more active in self-determined relative to predetermined finger-tapping sequences [59] and in tasks involving taking first-person perspective [24] and introspection [8]. The right SFC has been associated with self face recognition, mental-state attribution [36] and ‘Theory of Mind’ tasks, which require self- and other-perspective taking [23]. Finally, in a PET study by Fink and colleagues[60], which manipulated the congruency between the subjects' intentional movements and their visual sensory outcomes, the right SFC showed increased response to the conflicting situation in which intention and outcome did not match. Taken together, these studies show that the bilateral SFC is involved in self other judgments based upon movement. This concurs with our results of higher activity in these regions for the “responded self” condition than for the “responded other” condition.

#### Inferior Parietal Cortex

The IPC has been recognized as a prominent area of task-related deactivations [29], [32], [35]. In the present study, the IPC regions, especially on the right side, showed significant activation differences between the responded self and responded other conditions. This region appears to be involved in processes of integration between agency and body ownership and monitoring of discrepancies between intended and observed action [61]–[64], and has been recently implicated in voluntary decisions tasks, particularly in the right hemisphere [65].

Lesion studies have shown that damage to the IPC, especially on the right side, is associated with misattributions of limb ownership, with the patient perceiving his or her limb as an alien object or as belonging to another person [66]–[71]. Furthermore, schizophrenia patients who experience delusions of alien control show hyperactivity of the right IPC compared to patients with no such delusions [72], [73]. In normal subjects, IPC was shown to be involved in segregation of self from other in the body and movement domains: the right IPC showed more activity when participants imagined another person performing an action than when they imagined themselves acting. The opposite result was found for the left IPC, where the first-person perspective was associated with higher activation than the third-person perspective [43].

Similar activations of the IPC were observed in a task that required subjects to attribute a viewed motion to self vs. other based on movement-feedback congruence, which was manipulated by introducing an angular bias between the subject's motion and the corresponding visual feedback. The extent of the discrepancy between the movement and visual feedback was positively correlated with activity in the IPC as measured using PET [61]. Similar results were obtained when incongruence was manipulated in the temporal rather than in the spatial domain using fMRI [62].

Causal relationship between the IPC and self recognition has been tested using Transcranial Magnetic Stimulation (TMS). rTMS over the right IPL significantly impaired the subjects' performance in discriminating self faces from other faces [74], while rTMS over the left IPL impaired detection of asynchrony between actual and virtual hand movements when these movements were self generated [75]. Finally, direct cortical stimulation of the right angular gyrus in an epileptic patient induced illusionary body transformations and out-of-body experiences [76].

#### Precuneus

The precuneus is a “hot spot” of resting state metabolic activity and shows significant task-related deactivations when engaged in goal directed actions [77]. In the current experiment we found significant activity differences for the responded-self vs. responded-other conditions in this region. Recent experiments using fMRI and PET have shown this region to be active in tasks related to visuo-spatial imagery [78], [79], spatial attention [80], episodic memory [81], [82] and self-related processing [24], [27], [43], [63], [83], [84]. It has been suggested that the precuneus is involved in several aspects of self processing including judgment of self-descriptive traits [27], [83], [84], self-other perspective taking [23], [24], [43] and experience of agency [63]. These self-related functions are in line with the proposed role for the precuneus in episodic memory and visuo-spatial imagery and attention, as they may allow us to imagine our and other perspectives as well as connect our self schema with past knowledge. Furthermore, this region has been shown to be deactivated during slow wave sleep, REM sleep, hypnotic sate, general anesthesia and persistent vegetative state, all states of consciousness associated with an altered sense of self [77].

### Agency

By contrasting between the active and passive blocks, we aimed at localizing the processes related to agency and in particular, the neural signals related to self- initiated movement also known as “efference copy”. We found localized activity centered around the right central sulcus and thalamus as well as in the left cerebellum (see Supplementary Table S1). These activations correspond to the primary and secondary motor and somatosensory regions of the contralateral hand performing the action. Thus, they may simply reflect the motor and somatosensory activations corresponding to the movement itself. However, they may also be related to efference copy generation. Several PET studies have contrasted active and passive movements and reported differential activity in the contralateral MI, premotor cortex and SMA in the cerebellum alone [85] or no significant difference at all [86]. These inconsistencies may be attributed to the low temporal resolution of PET and fMRI - even using fast event-related designs as we did - does not allow detection of the transient neural traces involved in such processes.

### Regions outside the intrinsic system

While not the primary focus of this paper, certain regions outside the intrinsic system showed preferential activation in the responded self relative to the responded other condition (see Supplementary Table S1). In particular, the left insula, which showed such activity in the current study, has also been associated with self-relevant processing in two previous studies that contrasted an emotional valence task with a perceptual decision task [6], [87].

### Conclusion

The results of the current experiment are compatible with the notion of a functional division of the brain into two global systems, one that is oriented towards the external environment and the other that is tuned inward and deals with processing of self-related representations. Thus, our awareness appears to alternate between processing of stimuli impinging upon us from the outside and updating of our self representations in regard to those stimuli. Body representation requires that a balance be struck between these two processes, where the delineation of my body versus another's is based upon the sensations rising from external cues.

## Supporting Information

Figure S1 Intrinsic ROIs and Self Recognition - Left Hemisphere. A. Folded and inflated views of LH. B. Conjunction map of all tasks vs. fixation contrast. Multi-subject analysis (N = 11) testing whether at least one contrast activated the region in at least u subjects with FDR <0.05. The intensity represents the minimum number of subjects for whom the region was activated ranging from at least 4 subjects (orange) to 11 subjects (yellow). Graphs show multi-subject average BOLD activations of ROIs for responded self and responded other conditions. Intrinsic ROIs showed higher levels of activation in the responded self than in the responded other condition. Error bars represent SEM. * p<0.05 ** p<0.01
(10.19 MB TIF)

Figure S2 Extrinsic control regions and Self Recognition - Left Hemisphere. A. Folded and inflated views of LH. B. Conjunction map of all tasks vs. fixation contrast. Multi-subject analysis (N = 11) testing whether at least one contrast activated the region in at least u subjects with FDR <0.05. The intensity represents the minimum number of subjects for whom the region was activated, ranging from at least 4 subjects (orange) to 11 subjects (yellow). Graphs show multi-subject average BOLD activations of ROIs for responded self and for responded other conditions. Extrinsic control regions showed no difference in levels of activation between the responded self and responded other conditions. Error bars represent SEM. * p<0.05 ** p<0.01
(10.19 MB TIF)

Figure S3 BOLD activations in the Intrinsic ROIs by movement authorship. Average BOLD activations in the ROIs show no differences between the Active and Passive conditions. Error bars =  SEM.
(0.24 MB TIF)

Table S1 Regions of significant activity by condition. Agency (Active > Passive). Minimum cluster size 50 voxels. p<0.005 uncorrected for multiple comparisons at the voxel. All tasks ((Active+Passive)< Rest). Minimum cluster size 800 voxels. p<0.005 corrected for multiple comparisons. Self Recognition (Hits + False Alarms) <(Correct Rejections + Misses). Minimum cluster size 100 voxels. p<0.015 uncorrected for multiple comparisons at the voxel.
(0.08 MB DOC)
