# Supplementary figures and images for: Involvement of the Intrinsic/Default System in Movement-Related Self Recognition
Source: PLoS One. 2009 Oct 21;4(10):e7527. doi: 10.1371/journal.pone.0007527 (PMC2760765; doi:10.1371/journal.pone.0007527)

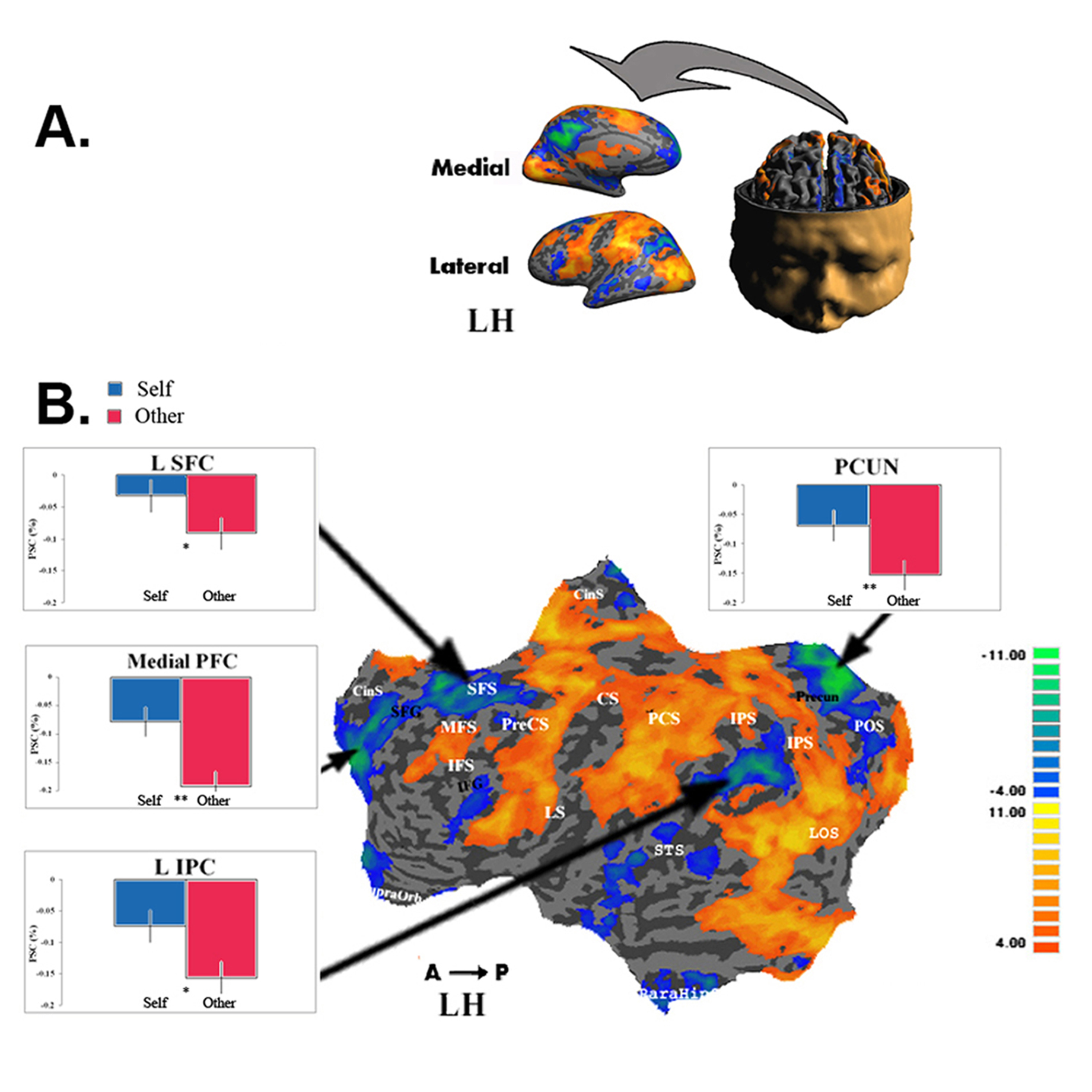

Supplement: Figure S1 — Intrinsic ROIs and Self Recognition - Left Hemisphere. A. Folded and inflated views of LH. B. Conjunction map of all tasks vs. fixation contrast. Multi-subject analysis (N = 11) testing whether at least one contrast activated the region in at least u subjects with FDR <0.05. The intensity represents the minimum number of subjects for whom the region was activated ranging from at least 4 subjects (orange) to 11 subjects (yellow). Graphs show multi-subject average BOLD activations of ROIs for responded self and responded other conditions. Intrinsic ROIs showed higher levels of activation in the responded self than in the responded other condition. Error bars represent SEM. * p<0.05 ** p<0.01 (10.19 MB TIF) [file pone.0007527.s001.tif]

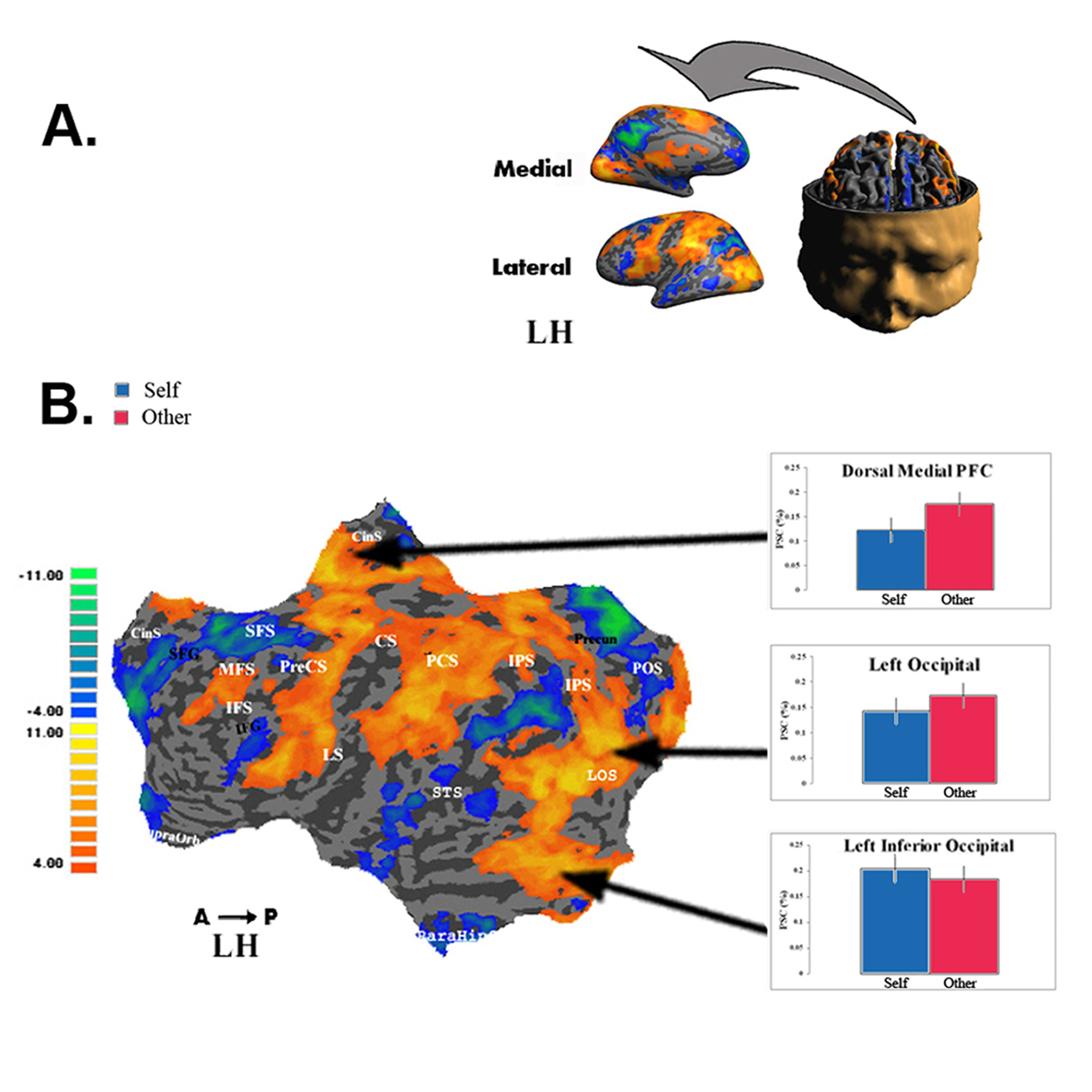

Supplement: Figure S2 — Extrinsic control regions and Self Recognition - Left Hemisphere. A. Folded and inflated views of LH. B. Conjunction map of all tasks vs. fixation contrast. Multi-subject analysis (N = 11) testing whether at least one contrast activated the region in at least u subjects with FDR <0.05. The intensity represents the minimum number of subjects for whom the region was activated, ranging from at least 4 subjects (orange) to 11 subjects (yellow). Graphs show multi-subject average BOLD activations of ROIs for responded self and for responded other conditions. Extrinsic control regions showed no difference in levels of activation between the responded self and responded other conditions. Error bars represent SEM. * p<0.05 ** p<0.01 (10.19 MB TIF) [file pone.0007527.s002.tif]

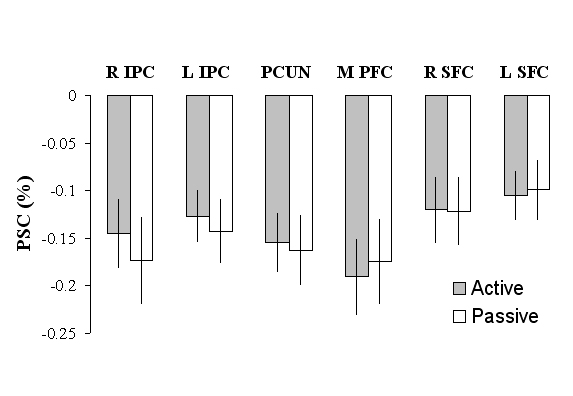

Supplement: Figure S3 — BOLD activations in the Intrinsic ROIs by movement authorship. Average BOLD activations in the ROIs show no differences between the Active and Passive conditions. Error bars = SEM. (0.24 MB TIF) [file pone.0007527.s003.tif]
