# Supplementary material for: Involvement of the Intrinsic/Default System in Movement-Related Self Recognition
Source: PLoS One. 2009 Oct 21;4(10):e7527. doi: 10.1371/journal.pone.0007527 (PMC2760765; doi:10.1371/journal.pone.0007527)
Supplement: Table S1 — Regions of significant activity by condition. Agency (Active > Passive). Minimum cluster size 50 voxels. p<0.005 uncorrected for multiple comparisons at the voxel. All tasks ((Active+Passive)< Rest). Minimum cluster size 800 voxels. p<0.005 corrected for multiple comparisons. Self Recognition (Hits + False Alarms) <(Correct Rejections + Misses). Minimum cluster size 100 voxels. p<0.015 uncorrected for multiple comparisons at the voxel. (0.08 MB DOC) [file pone.0007527.s004.doc]

**Table S1. Regions of significant activity by condition.**

| **Agency** | Side | X | Y | Z | Voxels | average t | average p |
| --- | --- | --- | --- | --- | --- | --- | --- |
| Cerebellum | L | -32 | -72 | -31 | 74 | 3.84 | 0.003 |
| Thalamus | R | 13 | -18 | 4 | 151 | 4.13 | 0.003 |
| Precentral Gyrus (BA 44) | R | 27 | -18 | 45 | 120 | 3.69 | 0.004 |
| Precentral Gyrus (BA 40) | R | 36 | -26 | 55 | 197 | 3.75 | 0.004 |
| Postcentral Gyrus (BA 31) | R | 48 | -21 | 59 | 78 | 3.93 | 0.003 |

| **All tasks** | Side | X | Y | Z | Voxels | average t | average p |
| --- | --- | --- | --- | --- | --- | --- | --- |
| Inferior Parietal Lobule (BA 40) | L | -46 | -37 | 34 | 12157 | 4.17 | 0.003 |
| Cingulate Gyrus (BA 24) | L | -16 | 0 | 50 | 14406 | 4.26 | 0.003 |
| Angular Gyrus (BA 39) | L | -48 | -64 | 31 | 2576 | -4.53 | 0.002 |
| Precentral Gyrus (BA 44) | L | -48 | 2 | 9 | 1844 | 4.53 | 0.002 |
| Middle Occipital Gyrus (BA 19 | L | -42 | -72 | 2 | 6712 | 4.02 | 0.003 |
| Cerebellum- Culmen | L | -30 | -54 | -18 | 1505 | 3.78 | 0.004 |
| Insula (BA 13) | L | -31 | 17 | 13 | 823 | 4.14 | 0.003 |
| Superior Frontal Gyrus (BA 6) | L | -17 | 25 | 52 | 2162 | -4.12 | 0.003 |
| Lentiform Nucleus | L | -23 | -5 | 20 | 2105 | 4.01 | 0.003 |
| Cerebellum- Pyramis | L | -5 | -66 | -26 | 835 | 3.82 | 0.004 |
| Cingulate Gyrus (BA 31) | L | 0 | -49 | 28 | 13982 | -5.61 | 0.001 |
| Superior Frontal Gyrus (BA 9) | L | -1 | 58 | 22 | 5687 | -4.28 | 0.002 |
| Cerebellum- Culmen | R | 1.9 | -56 | -9 | 4073 | 4.06 | 0.003 |
| Insula (BA 13) | R | 32 | 12 | 17 | 22389 | 4.48 | 0.002 |
| Superior Frontal Gyrus (BA 8) | R | 23 | 21 | 50 | 1587 | -4.16 | 0.003 |
| Parahippocampal Gyrus (BA 36) | R | 34 | -10 | -11 | 2873 | -4.25 | 0.002 |
| Fusiform Gyrus (BA 37) | R | 40 | -59 | -6 | 13120 | 4.52 | 0.002 |
| Superior Parietal Lobule (BA 7) | R | 25 | -59 | 45 | 2167 | 4.01 | 0.003 |
| Inferior Parietal Lobule (BA 40) | R | 50 | -31 | 32 | 5543 | 4.21 | 0.003 |

| ***Self Recognition*** | *Side* | *X* | *Y* | *Z* | *Voxels* | *average t* | *average p* |
| --- | --- | --- | --- | --- | --- | --- | --- |
| Precentral Gyrus (BA 4) | L | -56 | -5 | 18 | 296 | 3.33 | 0.009 |
| Sub-Gyral (BA 37) | L | -48 | -46 | -4 | 122 | 3.11 | 0.011 |
| Insula (BA13) | L | -45 | 2 | 5 | 122 | 3.14 | 0.011 |
| Fusiform Gyrus (BA 20) | L | -38 | -25 | -21 | 416 | 3.35 | 0.009 |
| Precentral Gyrus (BA 6) | L | -30 | 0 | 26 | 207 | 3.09 | 0.012 |
| Cingulate Gyrus (BA 24) | L | -21 | 5 | 45 | 120 | 2.99 | 0.013 |
| Precentral Gyrus (BA 4) | L | -19 | -26 | 56 | 162 | 3.10 | 0.011 |
| Posterior Cingulate (BA 30) | R | 10 | -50 | 19 | 142 | 3.00 | 0.013 |
| Paracentral Lobule (BA 6) | R | 14 | -27 | 47 | 2044 | 3.36 | 0.008 |
| Superior Frontal Gyrus (BA 10) | R | 8 | 62 | 22 | 293 | 3.15 | 0.010 |
| Cingulate Gyrus (BA 24) | R | 23 | -12 | 39 | 1425 | 3.22 | 0.010 |
| Superior Frontal Gyrus (BA 8) | R | 31 | 24 | 52 | 333 | 3.11 | 0.011 |
| Postcentral Gyrus (BA 2) | R | 44 | -23 | 48 | 233 | 3.15 | 0.011 |
| Inferior Parietal Lobule (BA 40) | R | 44 | -29 | 41 | 107 | 3.06 | 0.012 |
|  |  |  |  |  |  |  |  |
